# Development and Perspectives of Thermal Conductive Polymer Composites

**DOI:** 10.3390/nano12203574

**Published:** 2022-10-12

**Authors:** Jiaqi Wang, Lin Hu, Wenhao Li, Yuge Ouyang, Liuyang Bai

**Affiliations:** 1 College of Chemistry and Materials Engineering, Beijing Technology and Business University, Beijing 100048, China; 2 College of Energy Engineering, Huanghuai University, Zhumadian 463000, China

**Keywords:** thermal conductivity, heat transfer, polymer composites, fillers

## Abstract

With the development of electronic appliances and electronic equipment towards miniaturization, lightweight and high-power density, the heat generated and accumulated by devices during high-speed operation seriously reduces the working efficiency and service life of the equipment. The key to solving this problem is to develop high-performance thermal management materials and improve the heat dissipation efficiency of the equipment. This paper mainly summarizes the research progress of polymer composites with high thermal conductivity and electrical insulation, including the thermal conductivity mechanism of composites, the factors affecting the thermal conductivity of composites, and the research status of thermally conductive and electrical insulation polymer composites in recent years. Finally, we look forward to the research focus and urgent problems that should be addressed of high-performance thermal conductive composites, which will provide strategies for further development and application of advanced thermal and electrical insulation composites.

## 1. Introduction

With the advent of the 5G era, electronic devices and electronic equipment are developing toward miniaturization, integration, and high energy density [1,2]. However, a large amount of heat will be generated when various devices operate at high-power density, and the continuous accumulation of this heat will cause the device temperature to rise, thus affecting and reducing the function and service life of the devices [3,4]. The high peak temperature of miniaturized components may cause the thermal failure of the system, which accounts for ~55% of all failures [5]. Therefore, the industry generally believes that the bottleneck of the future development of electronic products is not the hardware or heat dissipation design, but whether effective heat dissipation materials can be prepared to solve the thermal failure problem in the operation of modern electronic products. Polymers are widely used in electronic packaging, thermally conductive adhesives, and other fields because of their chemical stability, good insulation, and industrial production [6,7,8,9,10,11,12,13,14,15,16]. However, most of the polymer materials are poor thermal conductors with low thermal conductivity, generally between 0.1–0.5 W·m^−1^·K^−^^1^, which greatly limits their wider applications [17,18].

In view of the above problems, the development of polymer composites with high thermal conductivity to better serve the development of 5G technology has gradually become a major challenge in the research of related materials. At present, there are two ways to improve the thermal conductivity of polymer materials. The first one is to improve the intrinsic thermal conductivity of polymer by changing the structure and arrangement of polymer chains, such as designing and synthesizing polymers with large conjugated π bond systems and introducing electronic heat conduction [19,20,21]. The orientation of the molecular chain is improved, or the liquid crystal structure is introduced through the action of the external field (stretching and electric field), and thus the local order of the molecular chain is improved [22,23]. However, because the modification of intrinsic polymers is concentrated on the micro scale, not only the process is complex, but the cost is high. Thus, it is difficult to popularize and apply on a large scale to enhance thermal conductivity greatly. Compared with it, the second and most effective way is to prepare filled polymer composites by adding high thermal conductive fillers, such as alumina, boron nitride, carbon fiber, and silver particles, into the polymer matrix [24,25,26,27,28,29,30,31]. During the past few decades, numerous efforts have been devoted to designing and fabricating polymers and polymer composites with high thermal conductivity [13,32,33,34,35,36,37,38,39]. At present, though many advances have been made in the field, there are still lots of research works and bottlenecks to be addressed for future industrial applications. This paper will first focus on the heat transfer mechanism of polymer composites in Section 2. Then, the main factors affecting heat transfer and the current research status of heat transfer of filled composites will be mainly discussed in Section 3 and Section 4, respectively. Finally, we look forward to future challenges and provide new strategies for the research of new high-performance thermal conductive and electrical insulating composites to promote the further development of the electronic information industry, such as 5G technology.

## 2. Heat Transfer Mechanism of Filled Thermal Conductive and Electrical Insulating Polymer Composites

According to the theory of thermal dynamics, heat conduction in solid matter can be regarded as carried out through the thermal vibration of microscopic particles and the carriers of microscopic particles mainly include electrons, molecules, and phonons [22,40,41]. There are lots of free-moving electrons in the metal material, and the collision between the electrons can quickly transfer heat, thus the metal materials show high thermal conductivity. In inorganic nonmetallic materials, there are almost no electrons that can move freely. For inorganic nonmetallic materials with good crystallinity, the heat transfer mainly depends on the vibration of the lattice. The thermal energy is first transmitted to the atoms on the surface of the material. The temperature of these atoms rises and vibrates violently [40]. At the same time, the thermal energy is transmitted in the form of waves to the adjacent atoms with lower temperatures and weaker vibration at the same speed, thereby generating heat conduction [40]. Therefore, inorganic nonmetallic materials with high crystallinity not only have high thermal conductivity but also have good electrical insulation performance.

**Figure 1:**
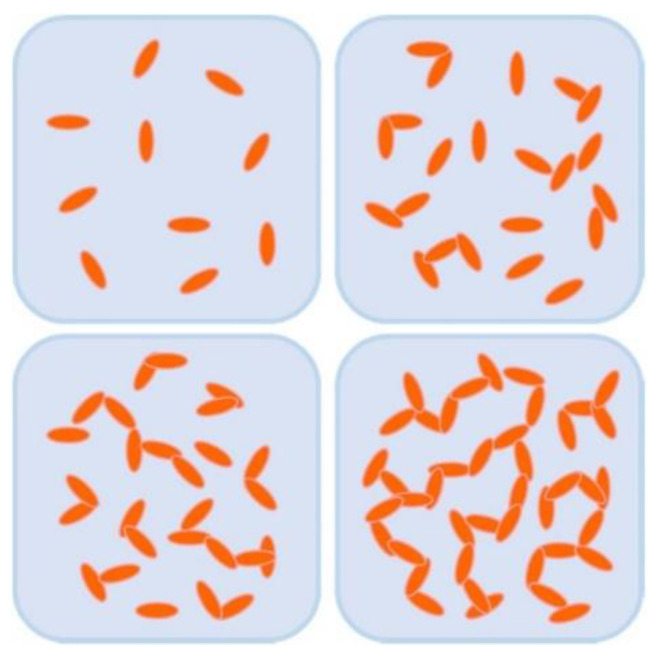
The distribution of filler particles in the polymer matrix. Reprinted with permission from Ref. [48]. Copyright 2018 Springer Nature.

Most of the polymer materials are saturated systems, which have no free-moving electrons inside, and mainly rely on phonons for heat transfer. It is the most effective way to improve thermal conductivity by adding high thermal conductivity fillers into the polymer matrix [25,42,43,44]. The thermal conduction mechanism of filled thermal conductive composites mainly includes the thermal conduction pathways theory, thermal conduction percolation theory, and thermal elastic coefficient theory [22,45,46,47], of which the thermal conduction pathways theory is most accepted and applied by researchers. According to this theory, the heat conductive fillers form continuous heat transfer pathways in the polymer matrix by contacting and overlapping each other, as shown in Figure 1 [48]. At a low addition, the filler particles are distributed in the matrix like “islands”, and thus the distance between them is too long to form effective heat transfer pathways, leading to a relatively low thermal conductivity of the composite. With the increase of the additional amount, the particles contact each other, and a more continuous heat transfer network structure is gradually built in the matrix. The internal heat of the composite will be efficiently transferred along the continuous heat conductive filler, thus showing a significantly improved thermal conductivity. This is similar to the percolation phenomenon of electrically conductive polymers, and thus some researchers believe that there is also a thermal percolation phenomenon in thermal conductive composites.

## 3. Factors Affecting Thermal Conductivity of Thermal Conductive and Electrical Insulating Polymer Composites

The thermal conductivity of polymer composites is affected by the polymer matrix and filler. The type of polymer matrix, filler type, loading, morphology, and interfacial adhesion will affect the final thermal conductivity of the composites.

### 3.1. Polymer Matrix

**Figure 2:**
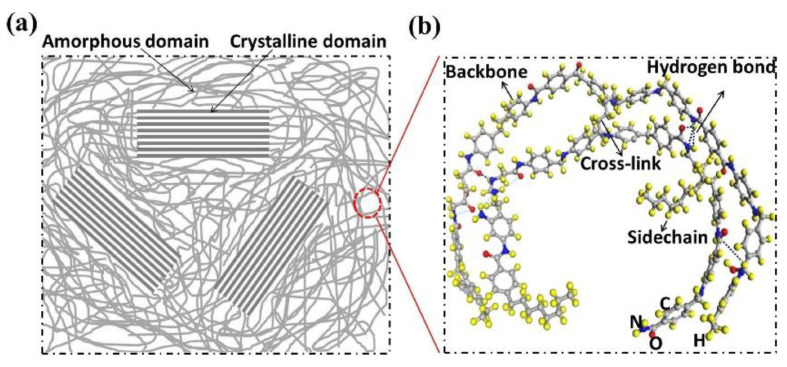
Schematic diagrams of a polymer: (**a**) the morphology of a polymer consisting of crystalline and amorphous domains; (**b**) structure of a polymer chain. Reprinted with permission from Ref. [49]. Copyright 2018 Elsevier.

A macromolecular block is a macrostructure formed by the winding and folding of many macromolecular chains which contains crystal regions and amorphous regions, as presented in Figure 2. The molecular chains in the crystal region are oriented in the same direction, arranged closely, and have periodicity, while the molecular structure in the amorphous region is disordered and has no periodicity.

In the amorphous region, irregular molecular chain arrangement leads to structural defects and interfaces, which will increase the phonons scattering and then lead to the low thermal conductivity of polymer bulk materials. The crystallinity, orientation, polarization degree of polar groups, number of atoms per unit volume, bonding strength, chain’s structure (including backbone bonds and side chains, and the inter-chain coupling), and crosslinking density of the material will all affect the intrinsic thermal conductivity. The theoretical thermal conductivity value of *λ* (W·m^−1^·K^−^^1^) can be calculated by the Debye formula (Equation (1)) [26,50]:

(1) $$\lambda =\frac{C_{P}vl}{3}$$

where $C_{P}$ is the specific heat capacity of the material, v is the phonon velocity, and l is the phonon free path. Table 1 lists the *λ* values of common polymer materials at room temperature [22,50,51,52,53,54,55,56,57,58,59,60].

The amorphous region can be converted into a crystal region by aligning the molecular chains to enhance the intrinsic thermal conductivity of the polymer material. The effective methods of aligning the molecular chains mainly include the mechanical stretching method, nano-template method, and electrospinning method [49].

The mechanical stretching method is to transform the amorphous structure of the polymer chain into a crystal structure with the same orientation through stretching, and effectively reduces the phonon scattering and improves the thermal conductivity by using the straight and ordered polymer skeleton and regular molecular structure. In 2010, Shen et al. [61] treated polyethylene nanofibers by mechanical stretching, and the thermal conductivity of the prepared nanofibers can reach about 104 W·m^−1^·K^−^^1^, which exceeded half of the pure metals including platinum, iron, and nickel, while the thermal conductivity of bulk polyethylene is usually in the order of 0.1 W·m^−1^·K^−^^1^. The realization of this high thermal conductivity depended on the improvement of the orientation of the polymer fiber chain by the stretching action, and the fiber quality further approaches the “ideal” polyethylene single crystal. Many breakthroughs have been made in the research of high thermal conductive polymer materials developed based on mechanical stretching strategies [62,63,64]. Xu et al. [65] used the mechanical stretching method to prepare a polyethylene film with a high tensile ratio and significantly enhanced thermal conductivity of about 62 W·m^−1^·K^−1^. The improvement of thermal conductivity under tensile action depended on the improvement of polymer chain orientation, molecular chain extension, molecular chain order, crystallinity, and crystal size.

The second common method is the nano-template, which is to melt the polymer material and penetrate the porous template to obtain the polymer nanofibers. When the melted polymer material flows into the nanopores, the molecular chain arrangement will become more orderly, thus the thermal conductivity of the nanofibers will be improved. Cao et al. [66] prepared high-density polyethylene (HDPE nanoarrays) with thermal conductivity of about 26.5 W·m^−1^·K^−1^ at room temperature using the improved nanoporous template wetting technology. The high orientation of the molecular chains of this nano array may be due to the combined effects of shear rate, vibration disturbance, translocation, nanoconfinement, and crystallization. This study provided more feasible directions for exploring how to improve the intrinsic thermal conductivity of polymers.

Electrospinning is a technology that uses electrostatic force to produce nanofibers. Polymer materials are firstly dissolved in organic solvents, and then the polymer solution is pulled out of the injection needle tip by electrostatic force to form polymer nanofibers [67]. During this process, most of the molecular chains in the nanofibers will be aligned with the axial direction, thus the thermal conductivity along the fiber direction can be significantly enhanced. In 2015, the thermal conductivity of electro-spun polyethylene nanofibers prepared by Ma et al. [68] at a voltage of 45 kV along the fiber axis was about 9.3 W·m^−1^·K^−1^, which was more than 20 times that of bulk polyethylene (about 0.4 W·m^−1^·K^−1^). The characterization results of micro-Raman spectra further confirmed the relationship between the influence of electrospinning on the orientation and crystallinity of polymer chains and the greatly improved thermal conductivity. The thermal conductivity of polycarbonate nanofibers prepared by Canetta et al. [69] by electrospinning was between 6.6 W·m^−1^·K^−1^ and 14.4 W·m^−1^·K^−1^, which was significantly improved compared with the thermal conductivity of polycarbonate bulk (0.15 W·m^−1^·K^−1^). In addition, Zhang et al. [70] studied the molecular weight and thermal conductivity of polymer electro-spun fibers and found that with the Mw of polyethylene fibers increasing from 35,000 to 3,000,000, the thermal conductivity continued to rise, which can be attributed to the higher crystallinity of high molecular weight polyethylene fibers.

### 3.2. Thermally Conductive Fillers

#### 3.2.1. Filler Type

Fillers commonly used in thermal conductive polymer composites include three types: metal fillers (silver, copper, aluminum), carbon-based fillers (carbon nanotubes, carbon fibers, graphene), and ceramic fillers (aluminum oxide, aluminum nitride, boron nitride) [2,17,25,26,71,72,73,74,75,76,77]. The values of thermal conductivity of various fillers at room temperature are shown in Table 2 [50,78,79,80,81,82,83,84,85,86,87,88,89,90,91,92]. Metal and carbon-based fillers play an important role in improving the heat transfer performance of polymer matrix due to their high intrinsic thermal conductivity. However, due to the high electrical conductivity of these two types of materials, the electrical insulation performance of the materials is often destroyed under high loading [81,93,94].For the thermal management materials applied between the chip set and the heat sink or soaking plate, not only high heat dissipation capacity but also good electrical insulation is required to avoid short circuits. Therefore, ceramic fillers with high thermal conductivity and high electrical resistivity possess more extensive applications in the field of thermally conductive and electrical insulating polymer materials.

#### 3.2.2. The Size and Morphology of Fillers

According to the heat conduction pathways theory, it is an effective way to improve the thermal conductivity of polymers by constructing a continuous filler network in the matrix. With the same loading, the size and morphology of the filler have an impact on the distribution of the filler in the matrix, which determines the continuity of the heat conduction channels.

**Figure 3:**
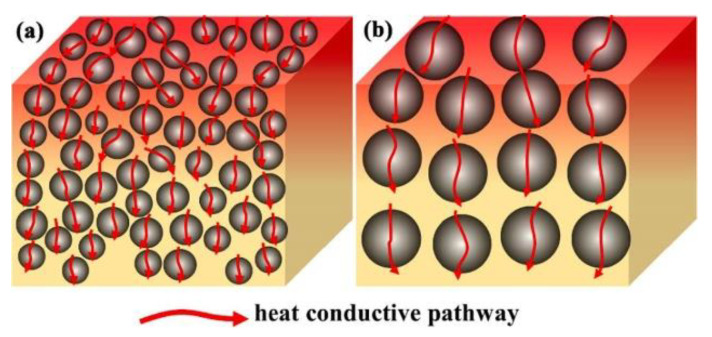
Heat transfer model of composites filled with (**a**) small particle size and (**b**) large particle size fillers. Reprinted with permission from Ref. [98]. Copyright 2022 Elsevier.

The particle size of the filler will affect the heat transfer inside the composite material to a great extent. At present, there is no conclusion about the influence of filler particle size on thermal conductivity. Firstly, in the view of forming heat conduction pathways, particles with smaller particle sizes are more conducive to heat transfer, because smaller particles can form more heat transfer pathways in a matrix, as shown in Figure 3a. Secondly, in view of interfacial thermal resistance, particles with small-size fillers possess a larger specific surface area, which will form more filler-matrix interfaces. When the carrier of phonon is transmitted in the composite material, phonon scattering will occur at the interface, resulting in interface thermal resistance. Therefore, the large particle size filler will reduce the area/amount of heat passing through the filler–-matrix interface, thus reducing the overall interfacial thermal resistance of the composites, as shown in Figure 3b. Different experimental results have been obtained on the effect of filler particle size on thermal conductivity. For example, Ren et al. [95] prepared spherical boron nitride with different particle sizes by spray granulation and applied them to polydimethylsiloxane. It was found that the particle with the size of 89 μm showed greater advantages in improving the thermal conductivity of the polymers. At the same loading of 50 wt%, the thermal conductivity of the composite reached 2.30 W·m^−1^·K^−1^, which was much higher than that of the particle size of 160 μm (about 1.75 W·m^−1^·K^−1^). The authors attributed this to the fact that the particles with smaller particle size exhibit higher particle packing density and promote heat transfer. However, Li et al. [96] studied the effects of boron nitride with various average particle sizes of 4 μm, 15 μm, and 27 μm on the thermal conductivity of polymers and found that the values of polypropylene composites increased with the increase of filler particle size under the same loading. The authors attributed this phenomenon to the fact that the filler particles with large particle sizes have a lower specific surface area and thus exhibit lower interfacial thermal resistance. In addition, the large-particle-sized particles were more likely to contact each other in the matrix to form a heat conduction network. The research results of Park et al. [97] showed that longer multiwalled carbon nanotubes were more efficient to improve the thermal conductivity of epoxy resin compared with shorter carbon nanotubes.

**Figure 4:**
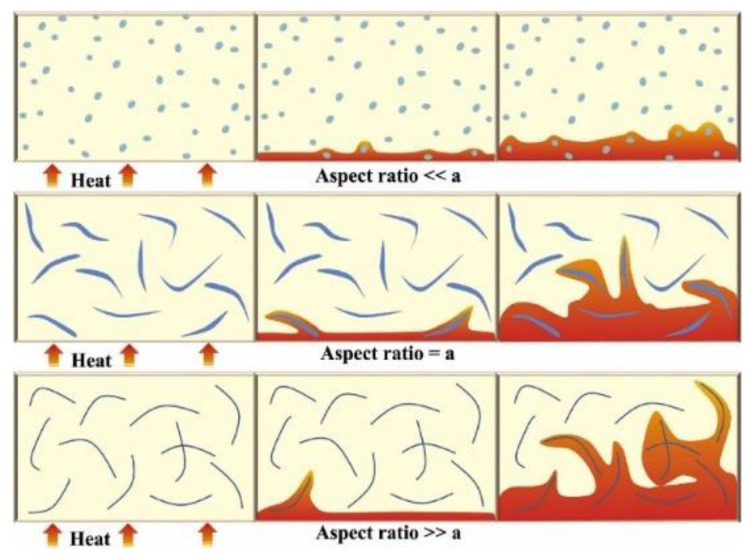
Internal heat distribution of polymer composites added with fillers with different aspect ratios. Reprinted with permission from Ref. [22]. Copyright 2020 Elsevier.

The morphology of fillers affects their distribution and contact state in the matrix. The aspect ratio of zero-dimensional spherical particles, one-dimensional fibrous or rod-shaped, and two-dimensional sheet fillers is different, and the filler network structure formed in the matrix is also various. It is generally believed that lamellar or fibrous fillers have a larger aspect ratio than spherical fillers and have fewer filler–-matrix interfaces in the length direction, which contribute to forming a continuous heat conduction network structure in the composite [40]. The heat transfer in composites filled with different aspect ratios was proposed by Guo et al. [22], as shown in Figure 4. The research results of Yu et al. [99] showed that with the increase of the aspect ratio of graphite nanosheets, the thermal conductivity of prepared epoxy resin composites gradually improved. When the loading was 5 vol%, the thermal conductivity of epoxy resin increased to 1.45 W·m^−1^·K^−1^ with graphite nanosheets with a high aspect ratio (GNP-800), which was much higher than that of the composites (0.54 W·m^−1^·K^−1^) with flake graphite fillers with the lowest aspect ratio (L~30 µm, t~10 µm). Therefore, the preparation of two-dimensional fillers with high aspect ratio and their application in thermal conductive composites have attracted a lot of attention in recent years [5,100,101]. In addition, Yan et al. [102] prepared the flake boron nitride nanolayer by microfluidic technology and used it to improve the thermal conductivity of polyvinyl alcohol. When the loading amount was 83 wt%, the thermal conductivity of the composite was increased to 67.6 W·m^−1^·K^−1^, which was about 355 times higher than that of the pure matrix.

Although fibrous or sheet-like fillers with high aspect ratios have obvious advantages in improving the thermal conductivity of polymer composites, such fillers cannot exert the best thermal conductivity if they are distributed in the matrix randomly and undirectionally. Therefore, it is necessary to apply additional methods to induce the fillers to conduct directional orientation in the composites during processing. In contrast, the composites filled with spherical particles display isotropic thermal conductivity. In addition, spherical fillers with smooth surfaces exhibit a relatively high viscosity percolation threshold (~50–60 vol%) in the polymer matrix, and thus composite with higher loading can be prepared without damaging the processability of the materials [81]. The irregular shape of filler particles will increase the friction with the matrix due to the edges, which will increase the viscosity of the mixture and lead to a decrease in the machinability and mechanical properties of the composite. Yeo et al. [103] introduced alumina particles with an average particle size of 45 µm to epoxy resin and found that the epoxy composite filled with spherical particles always had higher thermal conductivity with the same loading. The author attributed this to the fact that spherical particles had a smoother surface and lower specific surface area than irregular particles, which weakened phonon scattering during heat transfer.

#### 3.2.3. Surface Treatment of Fillers

When the thermal conductive fillers were introduced into the matrix, a large number of filler-matrix interfaces will be generated. When the heat is transmitted at the interfaces, the heat transfer efficiency will be reduced due to phonon scattering. In addition, due to the difference in polarity between the filler and the matrix, it shows poor compatibility. Therefore, improving the interface compatibility between the two is a common means to reduce phonon scattering. In recent decades, researchers have used different methods to treat the surface of fillers to improve the interfacial adhesion between fillers and polymer matrix [13,104,105,106,107,108,109,110,111,112,113]. Commonly used modifiers include surfactants, coupling agents, functional polymers, and inorganic coatings [81]. Surface modification not only improves the surface compatibility between filler and matrix but also improves the dispersion of particles, especially for nanofiller. In addition, based on the improvement of the interface adhesion performance, the internal defects of the material will be reduced, thus the mechanical properties and thermal stability of the composite will also be enhanced.

Common surface modifiers for inorganic ceramic fillers include silane coupling agents and dopamine. Ruan et al. [93] used dopamine to modify alumina particles and used it to prepare nitrile rubber composite. It was found that the thermal conductivity of the composite (0.211 W·m^−1^·K^−^^1^) was 122% higher than that of the pure matrix and slightly higher than that of the unmodified alumina-filled resin (0.204 W·m^−1^·K^−1^) when the addition amount was 30 phr, and the tensile strength of the composite was also increased by 255%. Bian et al. [114] used dopamine and KH550 to modify micron boron nitride and nano alumina, respectively, and the results showed that the thermal conductivity of the epoxy composite increased by 700% (1.182 W·m^−1^·K^−^^1^) when the amount of boron nitride and alumina was 22.5 wt% and 7.5 wt%, respectively. Carbon-based fillers, such as carbon nanotubes and graphene, are easy to agglomerate, thus acid treatment is often used for surface modification, which not only improves the dispersion of fillers in the matrix but also improves the mechanical properties of materials. However, this modification method often destroys the original structure of carbon material and reduces its crystallinity, which decreases the inherent thermal conductivity of the filler [22,40]. The research of Hong et al. [115] showed that the thermal conductivity of the acid-treated carbon-nanotube-filled polymethylmethacrylate composite was significantly lower than that of the untreated carbon-tube-filled composite. Yang et al. [116] found that the multiwalled carbon nanotubes modified by benzoic acid were well dispersed in the epoxy matrix and had better bonding performance with the interface. When the loading amount was 5 vol%, the thermal conductivity was increased by 684% compared with the pure matrix and about 65% compared with the untreated carbon-tube-modified epoxy resin composite. In this work, the author also studied the effect of acid-treated carbon nanotubes on the thermal conductivity of epoxy materials. It was also found that the thermal conductivity of the composites filled with acid-treated carbon nanotubes increased slightly and was lower than that of the composite modified with untreated fillers. The author attributed this to the increase in the defects on the side walls of carbon nanotubes after acid treatment, which hindered the heat transfer between phonons.

Although the surface modification of fillers improves the dispersion of fillers in the matrix and the interface compatibility with the matrix, which further improves the thermal conductivity to a certain extent. However, the modification process is complex, the conditions are harsh, and the environment is not friendly. In addition, the inherent thermal conductivity of most organic modifiers is low, and too much or too little addition will lead to interface defects and reduce the thermal conductivity of composites [81].

#### 3.2.4. Dispersion and Orientation of Fillers

The dispersion state of filler particles in the matrix directly affects the continuity of heat conduction channels. Most researchers believe that the better the dispersion of filler particles, the more favorable it is to build more and more continuous heat transfer channels in the matrix [13,18,105,117,118,119]. The surface treatment of filler particles can not only improve the compatibility between filler and matrix but also improve the dispersion of particles, thus effectively improving the heat transfer efficiency. However, like the influence of filler particle size on the thermal conductivity of composites, researchers have different views on the influence of filler dispersion on thermal conductivity. Guo et al. [22] believed that when the filler was uniformly dispersed in the matrix, the polymer molecular chain with low thermal conductivity was distributed around the particles, which hindered heat transfer. On the contrary, when the filler particles were properly agglomerated, a local heat transfer pathway can be formed, which will help to improve the thermal conductivity of the composites [22]. Burger et al. [40] added 7 wt% of expanded graphite and 3 wt% of different carbon black into epoxy resin and found that the thermal conductivity of resin filled with carbon black with large aggregates was higher than that of the composite added with uniformly dispersed carbon black particles. The authors emphasized that when the fillers were agglomerated, the distance between the particles decreased, and the fillers responsible for heat transfer improved the heat transfer efficiency. The results of Tanimoto et al. [120] showed that strongly aggregated particles can effectively transfer heat to neighboring particles and thus promoted the increase of thermal conductivity.

**Figure 5:**
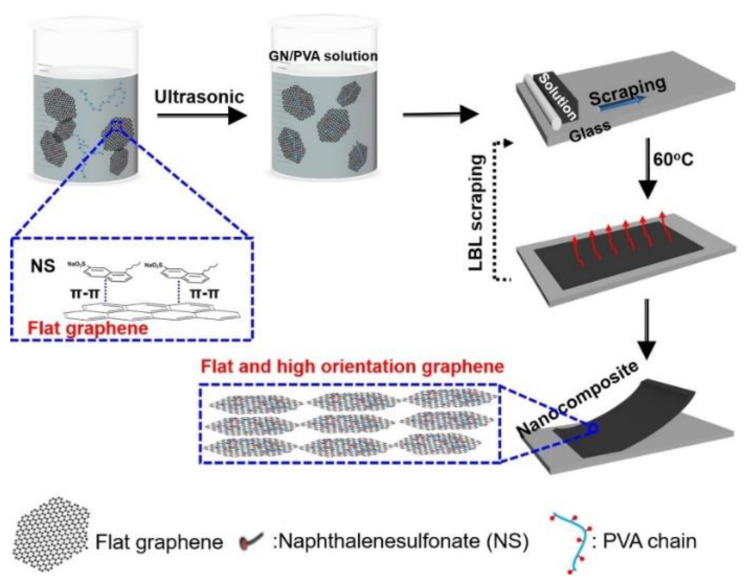
Preparation process of graphene-filled polyvinyl alcohol composite. Reprinted with permission from Ref. [101]. Copyright 2020 American Chemical Society.

Fillers with high aspect ratios, such as one-dimensional carbon nanotubes and carbon fibers, two-dimensional flake boron nitride and graphite, have anisotropic thermal conductivity. Therefore, when applied into polymer materials as fillers, they also exhibit anisotropic properties. Composites with high heat transfer performance in a specific direction are also required in specific applications and fillers with high aspect ratio can be aligned in the matrix through different preparation processes, such as hot pressing, injection molding, extrusion, external field (force field, magnetic field) and chemical vapor deposition [22]. Hence, the thermal resistance of the filler-matrix interface decreases, and the material exhibits high thermal conductivity along the direction of filler orientation (higher than the random distribution of fillers). Whereas, the thermal conductivity of the composite is relatively low in the direction perpendicular to the orientation of fillers [81]. Zhuang et al. [101] prepared graphene-filled polyvinyl alcohol nanocomposites with a high orientation by layer scraping method, and constructed π bonds between graphene layers, as shown in Figure 5. It was found that the in-plane thermal conductivity of the composites (in the graphene orientation direction) reached 13.8 W·m^−1^·K^−1^ and the thermal conductivity perpendicular to graphene orientation was 0.6 W·m^−1^·K^−1^ when the loading was 10.0 wt%. Pan et al. [121] prepared epoxy resin composite filled with two-dimensional sheet-like alumina filler and silver particle by hot pressing method. The results showed that the fillers were randomly distributed in the composite without hot pressing, while the filler were identical distribution orientation inside the composite formed after hot pressing and showed higher thermal conductivity.

## 4. Strategies for Enhancing Thermal Conductivity of Polymer Composites

### 4.1. Thermal Conductive and Electrical Insulating Polymer Composites Introduced with Single Filler

**Figure 6:**
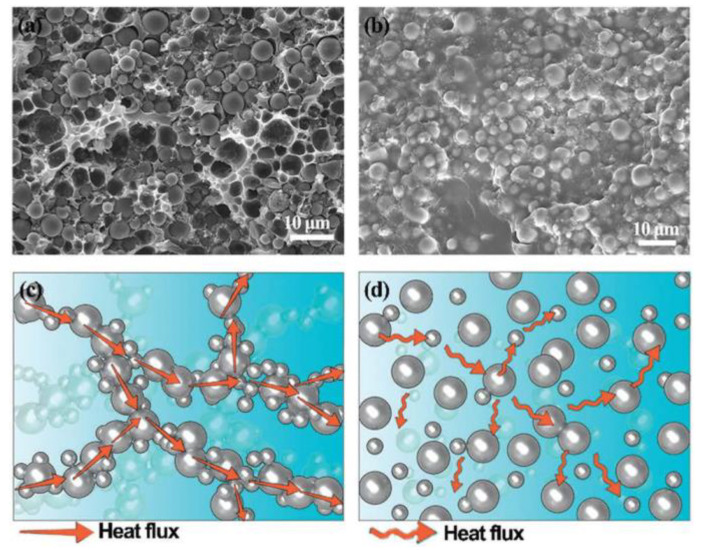
SEM images of alumina/polyvinyl alcohol composites prepared by (**a**) vacuum filtration and (**b**) simple mixing, internal heat transfer model of alumina/polyvinyl alcohol composites prepared by (**c**) vacuum filtration and (**d**) simple blending. Reprinted with permission from Ref. [123]. Copyright 2013 Royal Society of Chemistry.

The thermal conductivity of the composite filled with single filler is mainly affected by the inherent thermal conductivity of the filler, the loading, and the interfacial thermal resistance between fillers and matrix. Generally, the high inherent thermal conductivity of the fillers and high loading lead to better heat transfer performance of the composite. The composites filled with zero-dimension spherical or irregular fillers are isotropic, and generally require high loading to obtain continuous heat transfer pathways and achieve satisfactory thermal conductivity. However, a high loading will inevitably result in a large number of filler/matrixes interfaces and deteriorate the mechanical properties. As mentioned above, the problem can be solved to a certain extent by filling regular spherical particles and surface treatment. For fillers with a high aspect ratio or larger particle size, it is easier to overlap in the matrix to form continuous heat conduction pathways, which is more likely to achieve efficient phonon transmission pathways with relatively low loading. Wu et al. [122] prepared a polyvinyl alcohol composite with boron nitride as filler, and when the loading was 67.7 wt%, the thermal conductivity reached 10.04 W·m^−1^·K^−1^. Wu et al. [123] used spherical alumina as filler and constructed continuous alumina filler in polyvinyl alcohol by the vacuum filtration process. As shown in Figure 6, the thermal conductivity of the composite reached 4.79 W·m^−1^·K^−1^ when the content was 45.4 vol%, which was 2.33 W·m^−1^·K^−^^1^ higher than that of the composite prepared by simple blending.

### 4.2. Thermal Conductive and Electrical Insulating Polymer Composites Introduced with Hybrid Filler

In order to construct more continuous heat transfer pathways in a matrix at a lower loading, different types of fillers (two or more) are hybridized and filled into polymer matrices. The common hybridizations include fillers with different particle sizes and fillers with different types or morphologies [18,124,125,126,127,128,129,130].

#### 4.2.1. Hybrid Fillers with Different Particle Sizes

**Figure 7:**
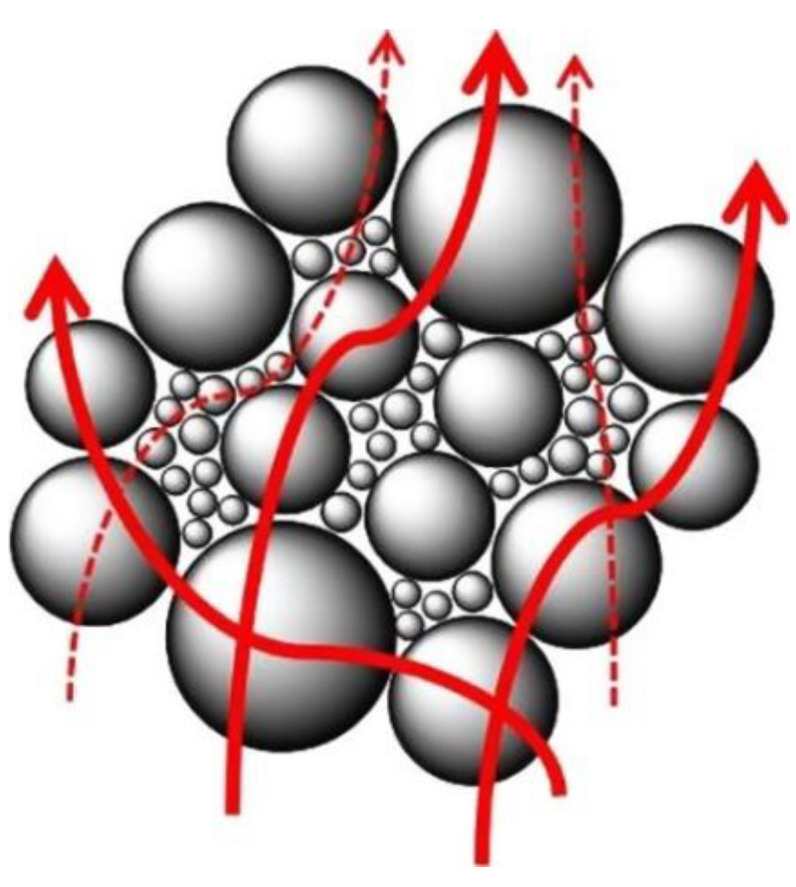
Heat transport model formed by fillers with different particle sizes. Reprinted with permission from Ref. [131]. Copyright 2013 Elsevier.

As discussed above, particles with large sizes reduce the thermal resistance between the filler and matrix, but the number of heat transfer channels formed is decreased. Small particle-size fillers can form more heat conduction pathways, but there are more interfaces that show high interfacial thermal resistance. Therefore, researchers consider hybrid fillers with different particle sizes, which can not only form more heat conduction pathways but also improve the packing density of fillers, and the heat transfer model as shown in Figure 7 [131]. The hybrid filler network shortens the distance between fillers, avoids heat passing through the matrix with low thermal conductivity, and promotes heat conduction. Li et al. [132] introduced hybrid spherical alumina with a diameter of 18 μm and 48 μm into nylon 6, and the influence of the ratio of different particle sizes on the thermal conductivity of the composite was systematically studied. It was found that the thermal conductivity of the prepared composite with hybrid fillers at various mass ratios was always higher than that achieved by a single particle size filler. The author attributed it to that the particles with small sizes distributed in the gap of the particles with large sizes, which increased the contact rate between the fillers, thus enriching the heat conduction pathways inside the material. Zhou et al. [133] also hybridized alumina with various particle sizes, and the prepared silicone rubber composite showed better heat transfer performance than the composites with single particle sizes. Chen et al. [134] hybridized spherical alumina with diameters of 30 μm and 5 μm into the epoxy matrix and the results showed that the thermal conductivity of the composite filled with hybrid filler is lower than that of the composite filled with single large particle size with the same addition amount but better than that of the composite filled with single small particle size filler. The authors explained that this is due to more interfacial scattering and higher interfacial thermal resistance caused by small particle size fillers. In addition, they also found that the viscosity of the system after the hybridization of the filler was reduced, the processability of the material is improved, and the prepared composite material has excellent mechanical properties, thermal stability, and electrical insulation at the same time.

#### 4.2.2. Hybrid Fillers with Different Types and Morphologies

**Figure 8:**
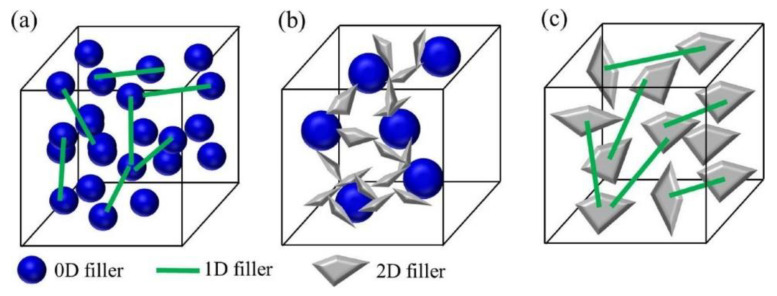
Schematic diagram of the network formed by the hybrid packings: (**a**) zero- and one-dimensional fillers, (**b**) zero- and two-dimensional fillers, (**c**) one- and two-dimensional fillers. Reprinted with permission from Ref. [49]. Copyright 2018 Elsevier.

Fillers with different intrinsic thermal conductivities and different aspect ratios can enhance the thermal conductivity of composites synergistically through hybridization. As shown in Figure 8, fillers with various morphologies form three-dimensional heat transfer pathways in the matrix through the “bridging effect”. Compared with the composite prepared by filling with a single filler, the hybrid network structure reduces the addition of filler and decreases interfacial thermal resistance, which promotes the construction of continuous heat transfer networks at a lower loading. Table 3 shows a summary of previous works on polymer composites with hybrid filler.

**Figure 9:**
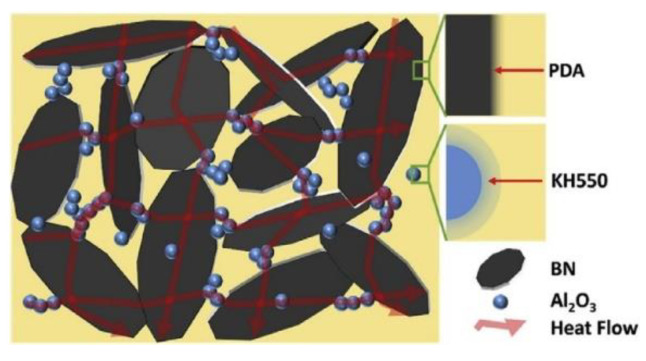
Heat transfer pathways formed by flake boron nitride and spherical alumina. Reprinted with permission from Ref. [114]. Copyright 2018 Elsevier.

Bian et al. [114] hybridized two-dimensional boron nitride and zero-dimensional alumina and filled the mixture into epoxy resin and found that the thermal conductivity reached 1.182 W·m^−1^·K^−1^ when the loadings of the two were 22.5 wt% and 7.5 wt%, respectively. The continuous heat transfer network formed in the matrix is shown in Figure 9. The author explained that the micron-scale boron nitride sheets form the main heat transfer pathways, and the nano-scale alumina forms a continuous phonon transfer path between the boron nitrides, thus the two synergistically improve the heat transfer efficiency of epoxy resin.

**Figure 10:**
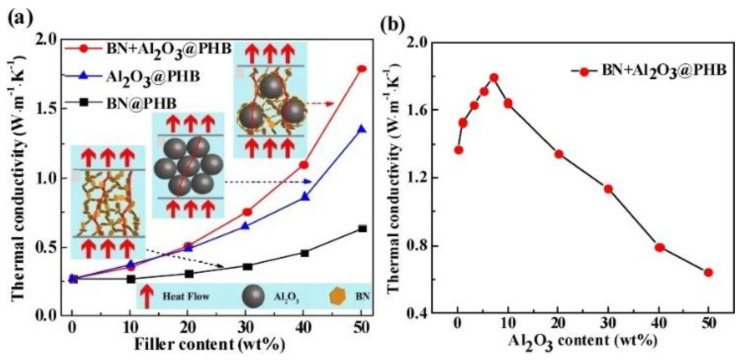
(**a**) Thermal conductivity and heat transport model of composites prepared from hybrid boron nitride and alumina, (**b**) effect of boron nitride and aluminum oxide ratio on thermal conductivity. Reprinted with permission from Refs. [49,137]. Copyright 2017 Elsevier.

Li et al. [137] also prepared poly(3-hydroxybutyric acid) composites by hybridizing two-dimensional boron nitride and zero-dimensional alumina. The study found that the maximum thermal conductivity achieved by the hybrid filler was 31% higher than that of the composite with single boron nitride and 196% higher than that of the single alumina particle filler, the results are shown in Figure 10. The study also showed that the boron nitride particles were distributed along the alumina surface when the alumina volume fraction is low, forming a network structure with a larger contact area. Therefore, the thermal conductivity gradually increased with the addition of alumina. When the content of alumina reached a critical value, boron nitride was distributed in the gaps between alumina particles, forming heat transfer pathways dominated by alumina, thus the thermal conductivity would show a downward trend.

Carbon materials, such as one-dimensional carbon nanotubes, two-dimensional graphite materials, and metal fillers, are also commonly used to hybridize with ceramic materials. Usually, the addition of a small amount of carbon materials or metal materials can effectively improve the thermal conductivity of composites and maintain the electrical insulation properties. Yu et al. [140] studied the hybrid filling of alumina and graphene into grease and found that the thermal conductivity of the grease reached 3.45 W·m^−1^·K^−1^ when the addition of graphene was only 1 wt%, which is much higher than the thermal conductivity (2.70 ± 0.10 W·m^−1^·K^−^^1^) without graphene. Pan et al. [121] hybridized a small amount of silver particles and two-dimensional alumina sheets to prepare an epoxy resin composite, which possessed greatly improved thermal conductivity and excellent electrical insulating properties when adding 0.6 wt% silver particles and 50 wt% alumina. Xue et al. [142] hybridized boron nitride and carbon nanotubes to prepare silicone rubber composites, and the results showed that the thermal conductivity of the composites filled with 0.25 vol% carbon nanotubes and 30 phr boron nitride was higher than that of the composite added with only 30 phr of boron nitride and even higher than the silicone rubber composite with 40 phr of boron nitride. At the same time, the electrical insulation of the composites was not deteriorated due to the introduction of carbon nanotubes, which the authors attributed to the low content of carbon nanotubes and the separation of carbon nanotubes in the matrix by boron nitride, which makes it difficult to form continuous electrically conductive pathways. Moreover, the embedded carbon nanotubes introduced electron traps that capture and confine the movement of charge carriers, which in turn hindered the improvement of electrical conductivity.

**Figure 11:**
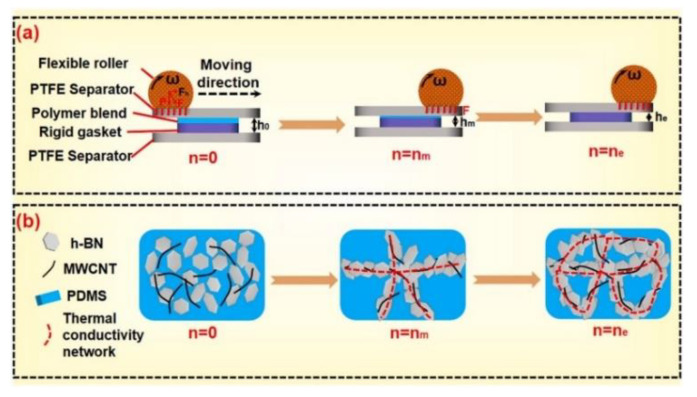
Illustration of (**a**) the CSNA method, (**b**) the forming process of the thermal conductive network of the hybrid filler in polydimethylsiloxane matrix. Reprinted with permission from Ref. [143]. Copyright 2022 Elsevier.

Fu et al. [143] constructed the network structure of hexagonal boron nitride and multiwalled carbon nanotubes in polydimethylsiloxane matrix by the continuous spatial confining forced network assembly (CSNA) method. The preparation process and the heat transfer pathways formed are shown in Figure 11. It is found that the thickness of the sample affects the thermal conductivity of the prepared composite. When the thickness of the sample is 0.15 mm and the loading of hexagonal boron nitride and multiwalled carbon nanotubes was 30 wt% and 2 wt%, respectively, the thermal conductivity of the composite reached 4.28 W·m^−1^·K^−1^, which is 1543.5% higher than that of the pure polydimethylsiloxane matrix. However, the electrical insulation of the material was decreased, and the volume resistivity was lower than the critical value of the insulation property of the material (10^9^ Ω · cm). In contrast, the composites filled with 1wt% multiwalled carbon nanotubes and 30 wt% hexagonal boron nitride displayed enhanced thermal conductivity and good electrical insulation performance. The above research results further confirmed that the additional amount of carbon-based or metal-based fillers will greatly affect the electrical insulation performance of polymer composites, which is an important factor to be considered by researchers and industry.

### 4.3. Construction of Continuous Filler Network to Prepare Thermal Conductive and Electrical Insulating Polymer Composites

Although the thermal conductivity can be improved more effectively by high loadings or hybridizing fillers with different particle sizes and morphologies; however, as the filler particles are not in contact with each other directly, the polymer matrix with low thermal conductivity between them hinders the formation of continuous heat transfer pathways. Therefore, researchers have managed to control the distribution of fillers through various effective means, and then construct a completely continuous filler network [25,38,71,72,77,145,146,147,148,149,150,151,152]. This approach not only directly reduces the number/area of the interface between filler and matrix but also reduces the filler–filler interface, which fundamentally decreases the interfacial thermal resistance during heat transfer. Common methods for building continuous thermal networks include gel-freeze-drying, the organic template method, and adding a pore-forming agent. For ceramic fillers, there are also high-temperature sintering methods [153,154,155,156,157]. Table 4 summarizes the thermal conductivity of polymer composites with continuous filler network structure.

**Figure 12:**
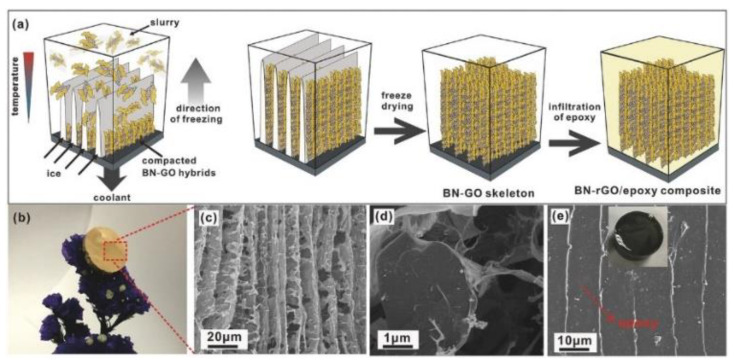
(**a**) Preparation process and (**b**) photograph of epoxy composites, (**c**,**d**) cross-sectional morphologies of boron nitride and reduced graphene oxide, and (**e**) cross-sectional morphologies of composites. Reprinted with permission from Ref. [153]. Copyright 2018 John Wiley and Sons.

Chen et al. [160] mixed boron nitride nanosheets and nanocellulose and prepared gel by epichlorohydrin crosslinking. Then, a three-dimensional interconnected porous boron nitride heat conduction network was obtained by the freeze-drying method. Finally, epoxy resin was impregnated by the vacuum-assisted method to prepare the composite. It was found that when the boron nitride loading was 9.6 vol%, the thermal conductivity of the nanocomposite reached 3.13 W·m^−1^·K^−1^, which was about 1400% higher than that of pure epoxy. Zeng et al. [159] applied boron nitride nanosheets as the basic assembly unit to prepare thin-film polyvinyl alcohol composites with microscopic structures similar to natural shells by vacuum-assisted suction filtration technology. The dense stacking of 2D boron nitride fillers formed a continuous thermal transport network, which enhanced the thermal conductivity of polyvinyl alcohol to 6.9 W·m^−1^·K^−^^1^. Yao et al. [153] prepared a hybrid network framework of boron nitride and reduced graphene oxide by freeze-drying and prepared epoxy composites by suction filtration. The preparation process and the microstructure of the materials are shown in Figure 12. It was found that the composite exhibited a high thermal conductivity of 5.05 W·m^−1^·K^−1^ at a relatively low loading of 13.16 vol%.

**Figure 13:**
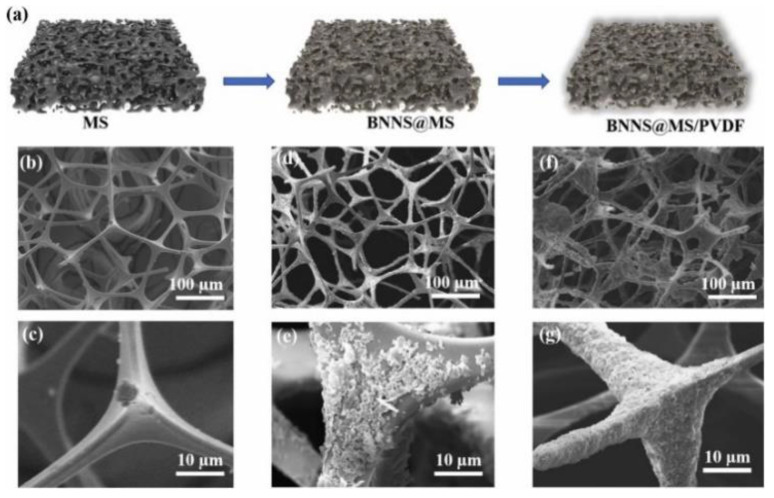
(**a**) Schematic illustration of BNNS@MS/PVDF, SEM images of: (**b**,**c**) MS, (**d**,**e**) BNNS@MS, and (**f**,**g**) BNNS@MS/PVDF. Reprinted with permission from Ref. [25]. Copyright 2022 Elsevier.

Lei et al. [25] used a melamine formaldehyde resin sponge (MS) as the skeleton (Figure 13) and successfully prepared the three-dimensional skeleton of the resin sponge wrapped by boron nitride nanosheets through the repeated layered assembly. Polyvinylidene fluoride (PVDF) was infiltrated into the framework of boron nitride by vacuum suction filtration process to prepare polyvinylidene fluoride (PVDF) composite, as shown in Figure 13. Due to the continuous phonon transport network constructed, the thermal conductivity of the composite reaches 1.43 ± 0.2 W·m^−1^·K^−1^ when the content of boron nitride was about 5 wt%, which is 51.1% higher than that of the randomly distributed boron-nitride-filled composite.

In addition, Hu et al. [156] and Xiao et al. [158] prepared alumina porous ceramics by high-temperature sintering and then prepared alumina/epoxy composites by vacuum filtration. In this method, the isolated particles were connected through the sintering neck formed by the sintering mass transfer of particles in the high-temperature environment, which reduced the interfacial thermal resistance at the filler-matrix interface and reduced the heat transfer resistance between the filler and filler, and thus effectively improves the heat transfer performance of the epoxy matrix.

## 5. Summary and Prospect

Thermal conductive and electrical insulation composites are widely used in electronic device packaging, high-power circuit substrates, and thermally conductive interface materials. This paper summarizes the heat transfer mechanism of the solid material, the main factors affecting the heat transfer performance of the composite, and the research progress of the polymer thermal conductive composite. On this basis, the following main conclusions can be drawn:

(1) In a polymer matrix, heat conduction mainly depends on phonon transport. The disordered structure of polymer molecular chains and the weak interaction between molecules lead to serious phonon scattering, which reduces the inherent thermal conductivity of polymer materials. The mechanical stretching method, nano-template method, and electrospinning method are the main methods to improve the inherent thermal conductivity of polymers. Although the inherent thermal conductivity of polymers can be improved by designing the degree of orientation, crystallinity, or molecular structure of polymer molecular chains, it is extremely difficult to achieve control of polymer molecular structures at the micro-scale.
(2) The preparation of thermally conductive polymer matrix composites by adding fillers with high thermal conductivity to the polymer matrix is an effective way to improve the thermal conductivity of polymer materials, which is currently the most studied and easy-to-realize large-scale production and application. The properties of fillers (such as particle size, morphology, dispersion, and orientation) and the interfacial compatibility between fillers and polymer matrix will affect the heat transfer efficiency of composites. Although the surface modification of filler particles can improve the interface compatibility between filler and matrix, thereby weakening the phonon scattering, it is still very limited in improving the overall thermal conductivity of materials.
(3) The polymer composites filled with various fillers (with different particle sizes, different morphologies, and different types) have higher thermal conductivity than those with single fillers. This is because one of the fillers is dispersed between the other fillers, connecting the other fillers like a bridge, and building a more continuous heat transfer path in the matrix, which reduces phonon heat dissipation and interface thermal resistance in the process of heat transfer. In addition, the small particle size fillers are dispersed in the gap of the large particle size fillers, which improves the packing density of the filler in the matrix and enrich the heat transfer pathways.
(4) Constructing a continuous filler network in the polymer matrix has attracted more research in recent years. The commonly used preparation methods include freeze-drying, skeleton preparation, and high-temperature sintering. The continuous network structure of building filler is more conducive to achieving higher thermal conductivity under lower load than the composite materials prepared by filling single or hybrid filler, which is not only conducive to achieving the lightness of materials but also will not reduce the mechanical properties and processability of composites. Therefore, this will be one of the main directions of future research.

Although great progress has been made in the field of high thermal conductivity polymer composites in the past decades, there are still many problems and challenges that should be addressed. We list several aspects that need to be studied in the future, as follows:

(1) In order to more accurately analyze the influence of fillers (particle size, dispersion, network structure, etc.) on thermal conductivity, more reliable theoretical models (considering particle size and morphology, etc.) should be established to predict the thermal conductivity, and simulation software such as COMSOL Multiphysics and ANSYS software should be used to analyze the whole process of heat transmission in materials.
(2) In-depth analysis of the micro-interface state in polymer composites, including the influence of filler-matrix bonding force, contact area, and contact morphology on the interfacial thermal resistance. Develop new technologies and new instruments that can accurately test the interfacial thermal resistance, and clarify the relationships between the specific surface area of the filler, the interface area between filler and matrix and the interfacial thermal resistance, which will provide a scientific basis for optimizing the two-phase interface and reducing the heat transfer resistance.
(3) Develop new ways to construct filler network structures in the matrix (not only limited to freeze-drying, vacuum-assisted filtration, etc.). The preparation process should be simple and cost-effective, which is conducive to large-scale production and practical application. At the same time, consider other characteristics of the composites, such as low loading, mechanical properties, long-term thermal stability, thermal expansion, flame retardancy, aging, etc.
(4) Broaden the new way of controllable synthesis of thermally conductive filler, and take boron nitride as an example, develop a new strategy for preparing boron nitride with a high aspect ratio, which is easy to operate and has a high yield ratio. The preparation of high thermal conductivity fillers with various morphologies and particle sizes is the premise and key to achieving low loading and high thermal conductivity.

## Figures and Tables

**Table 1:** *λ* values (W·m^−1·^K^−1^) of common bulk polymers.

Polymers	λ	Polymers	λ
High-density polyethylene	0.44	Poly(butylene terephthalate)	0.29
Low-density polyethylene	0.26	Epoxy resin	0.23
Polyphenylene sulfide	0.29	Polyetheretherketone	0.25
Polymethylmethacrylate	0.21	Polyvinyl chloride	0.21
Polyurethane	0.21	Nylon-6	0.28
Polyimide	0.27	Polyacrylonitrile	0.26
Phenolic resin	0.25	Polycarbonate	0.23
Polypropylene	0.21	Polystyrene	0.20

**Table 2:** *λ* values (W·m^−1^·K^−1^) of commonly used thermally conductive fillers.

Fillers	λ	Fillers	λ
Aluminum	247	Copper	398
Silver	427	Gold	315
Carbon nanotubes	1000–4000	Carbon fiber	300–1000
Graphite	100–400	Graphene	2000–6000
α-Alumina	36	Beryllium oxide	230–330
Cubic boron nitride	1000	Hexagonal boron nitride	300
Aluminum nitride	100–300	Silicon carbide	120

**Table 3:** Summary of *λ* values (W·m^−1^·K^−1^) of polymer composites filled with hybrid fillers.

Filler 1	Filler 2	Matrix	Loading	λ	Ref.
Graphene, 0.35 to 12 nm (thickness) 2 to 8 μm (lateral)	Hexagonal boron nitride 0.35 to 12 nm (thickness) 3 to 8 μm (lateral)	Epoxy	21.8 vol% + 21.8 vol%	6.50	[135]
spherical α-Alumina, (∼30 nm)	Hexagonal boron nitride (∼10 μm)	Epoxy	22.5 wt% and 7.5 wt%	1.182	[114]
Alumina, d50: 12.5 ± 5.35 μm	Boron nitride d50: 8.3 ± 5.20 μm	Epoxy	52 wt% and 10 wt%	1.65	[136]
Spherical Alumina	Hexagonal boron nitride	poly(3-hydroxylbutyrate)	7wt% and 43 wt%	1.79	[137]
Alumina, 3 μm	Boron nitride platelets, 32.52 μm	polydimethylsiloxane	30 wt% and 5 wt%	3.64	[138]
Graphene nanoplatelets with 15 μm lateral size and 10 nm thickness	Boron nitride nanoparticles (nm-BNs, 110 nm)	Epoxy	16 vol% + 1 vol%	4.72	[139]
α-Alumina, average particle sizes of 0.7 and 5 μm	Graphite oxide	Silicone oil	63 vol% and 1 wt%	3.45	[140]
Alumina flake, average thickness of 372 nm and a diameter of 8.2 μm	Silver particle, 10–20 nm	Epoxy	50 wt% and 0.6 wt%	6.71	[121]
Alumina, 4.98 μm	Graphene nanosheets, the average lateral size is 4.96 μm	Epoxy	42.4 wt% and 12.1 wt%	33.4 in radial direction and 13.3 in axial direction	[141]
Multiwalled carbon nanotubes with a length of 10 μm and a diameter of 11 nm	Boron nitride with a particle size of 1–2 μm	Silicone rubber	0.25 vol% and 30 phr	0.279	[142]
Hexagonal boron nitride	Multiwalled carbon nanotubes with a length of 3–12 μm	Polydimethylsiloxane	30 wt%+2 wt%	4.28	[143]
Hexagonal boron nitride with a lateral size of approximately 70 nm and a normalized thickness of 7 nm	Graphene oxide nanosheets have a normalized lateral size of 860 nm and a normalized thickness value of 1.6 nm	Poly(vinyl alcohol)	less than 1 wt%	9.9, in-plane	[130]
Graphene nanoplatelets, diameter, 1–20 mm; thickness, 5–15 nm	Alumina fibers with diameters ranging from 1.6 mm to 2.3 mm, several centimeters in length	Epoxy	2 vol% and 50 vol%	1.62	[129]
Boron nitride, diameter: 12 μm	Graphene oxide	Poly(dimethylsiloxane)	30 wt% and 2 wt%	10.91 in-plane and 1.27 through-plane	[127]
Alumina, 1 μm	Carbon fiber, the diameter is 10 μm and mean length is 150 μm	Silicone rubber	5 vol% and 25 vol%	9.60	[144]

**Table 4:** Summary of *λ* values (W·m−1·K−1) of polymer composites filled with continuous packing networks.

Filler	Matrix	Preparation Method	Loading	λ	Ref.
Boron nitride nanosheets	Poly(vinylidene fluoride)	Prefabricated skeleton and vacuum filtration	5 wt%	1.43 ± 0.2	[25]
Alumina (1–3 μm)	Epoxy	Prefabricated skeleton and sintering	23.32 vol%	2.58	[158]
Boron nitride nanosheets	Poly(vinyl alcohol)	Vacuum-assisted self-assembly	-	6.9	[159]
Boron nitride nanosheets	Epoxy	Sol–gel and freeze-drying	9.6 vol%	13	[160]
Boron nitride nanosheets	Epoxy	Ice-templated approach and filtration	9.29 vol%	2.85	[157]
Alumina powder with average particle size D50 = 1.36 μm	Epoxy	Sintering and vacuum filtration	70 vol%	13.46	[156]
Commercial alumina particles	Epoxy	Template method and vacuum filtration	36.2 vol%	3.17	[154]
Reduced graphene oxide	Silicone rubber	Prefabricated skeleton and vacuum filtration	1.46 wt%	1.50	[155]
Boron nitride platelets and reduced graphene oxide	Epoxy	Ice-templated and infiltrating	13.16 vol%	5.05	[153]
Boron nitride nanosheet	Polydimethylsiloxane	In-situ construction	9.82 vol%	0.94	[152]
MXene and graphene	Polyethylene glycol	Prefabricated skeleton and vacuum filtration	18.7 wt%	2.44	[24]
Alumina sphere	Phenolic resin	Sintering and vacuum filtration	75 wt%	4.01	[161]
Boron nitride nanosheets	Poly(p-phenylene benzobisoxazole)	Sol−gel-film conversion method	10 wt %	21.34	[162]
Boron nitride nanosheets	Polydimethylsiloxane	Sugar-templated method and vacuum filtration	25 vol%	7.55 in-plane and 1.12 through-plane	[163]
SiC nanowires	Silicone rubber	Prefabricated skeletons and template-assisted chemical vapor deposition method	15 vol%	2.13	[33]
